# Spectral Contributions to Circularly Polarized Luminescence beyond Artifacts in Solution-Processed Thin Films

**DOI:** 10.1021/acs.jpcc.6c05545

**Published:** 2026-09-11

**Authors:** Sithara R. Rao, Justin J. O’Neil, David H. Waldeck, Brian P. Bloom

**Affiliations:** Department of Chemistry, 6614 University of Pittsburgh, Pittsburgh, Pennsylvania 15260, United States

## Abstract

Circularly polarized luminescence (CPL) spectroscopy is a powerful tool used for probing the preferential emission of left and right circularly polarized light from chiral luminescent materials. Accurate interpretation of CPL spectra remains challenging, however, owing to relatively small signal intensity and artifact-laden responses along with linear anisotropy and birefringence effects associated with sample anisotropy. Herein, we demonstrate that additional spectral features become manifest in the CPL spectra of chiral perovskite thin films, an emerging material with potential in sensing, catalysis, data storage, and advanced display applications. CPL spectra show discrete transitions with varying sign and are attributed to the coexistence of crystalline perovskite, intermediate, and precursor phase domains formed during non-uniform crystallization, each contributing independently to the ensemble-averaged signal. These findings are corroborated by microspectrophotometry measurements, which directly resolve these spatially distinct domains and their characteristic absorbance profiles. Collectively, this work underscores the importance of accounting for coexisting emissive components when interpreting CPL spectra of solution-processed thin films.

## Graphical abstract

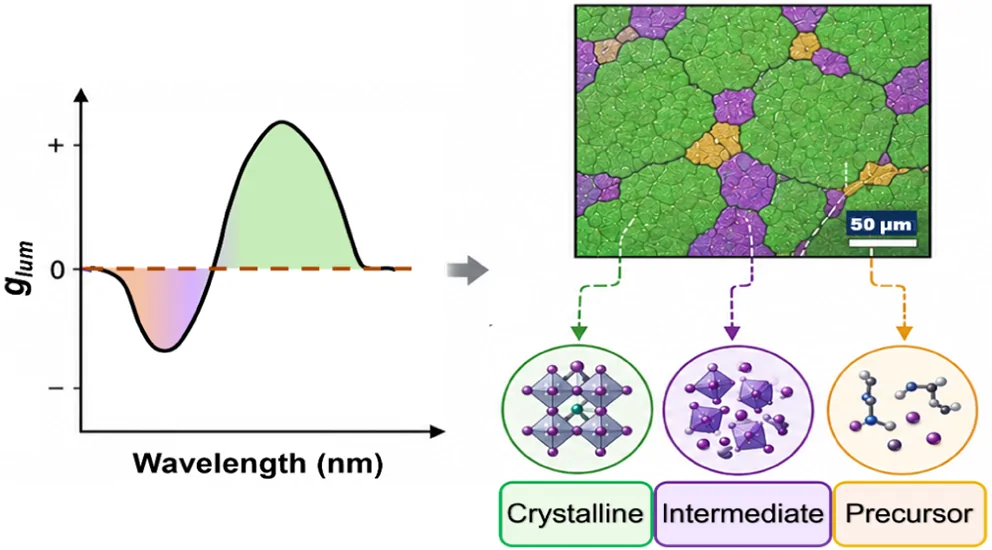

## 1 Introduction

Circularly polarized luminescence (CPL) corresponds to the non-zero differential emission of left and right circularly polarized light from chiral luminescent materials and is quantified by the dissymmetry factor, *g*_*lum*_:

$$g_{\mathrm{lum}}=2\left(I_{L}-I_{R}\right)/\left(I_{L}+I_{R}\right)$$

in which *I*_*L*_ and *I*_*R*_ correspond to the emission intensities of left- and right-handed circularly polarized light, respectively.^1,2^ CPL spectroscopy has been used to probe the excited-state chiroptical properties of a broad range of chiral systems, including chiral lanthanide complexes,^3,4^ nanomaterials,^5,6^ inorganic semiconductor films,^7,8^ conjugated polymers,^9,10^ small-molecule organic emitters,^11,12^ among others. The ability to generate and manipulate circularly polarized light has received considerable interest for applications in chiral sensing,^13^ asymmetric photocatalysis,^14^ optical data storage,^15^ and next-generation displays and communication technologies.^16,17^ The accuracy and reliability of CPL measurements are thus paramount for understanding the materials properties which give rise to large dissymmetry factors as well as for identifying materials suitable for a given application.

Isolating the “true” CPL of a material, however, remains challenging, as spectra are susceptible to artifact-induced signals that can distort and even invert the response.^18^ Misinterpretation can thus lead to incorrect conclusions regarding the excited-state chirality of a material, incorrect assignment of emissive states, and unreliable *g*_*lum*_ values that undermine the validity of structure-property relationships.^19–21^ Typically, artifacts in CPL measurements arise from the coupling between preferential photo-selection of parallelly oriented transition dipoles, fluorescence anisotropy (FA), and polarization distortion by birefringent materials, linear birefringence (LB), with the optical elements of the detection system.^22,23^ These effects are particularly prevalent in samples with restricted molecular motion, including viscous solutions, amorphous films, as well as highly oriented systems, e.g., single crystals and crystalline thin films, where macroscopic structural anisotropies introduced during sample preparation can generate false CPL signals that closely mimic genuine chiroptical responses.^24–26^ Strict standardization protocols and careful instrumental calibration are therefore essential to deconvolute genuine CPL from artifact contributions.^27,28^

Beyond measurement artifacts, the emergence of multiple distinct emissive regions in a material represents an equally important, yet often underappreciated source of spectral complexity in CPL measurements. Spatial non-uniformity in composition, crystallinity, and phase distribution is common in solution-processed organic, inorganic, and hybrid thin films, and can give rise to diverse emissive components within a single film, each carrying its own spectroscopic signature.^29–33^ The collective contribution of these components to photoluminescence and CPL spectra can therefore produce complex line shapes that are difficult to interpret without spatially resolved characterization. Herein, we perform CPL measurements on chiral perovskite thin films to show such coexistence of spectrally distinct emissive domains giving rise to a bisignate spectral line shape.

Chiral two-dimensional (2D) hybrid perovskites, in which chiral organic cations are incorporated into a perovskite framework, have emerged as a promising class of chiroptical materials owing to their strong CPL responses,^34–36^ tunable optical properties,^37,38^ and solution processability.^39,40^ Moreover, incorporation of dopants into chiral perovskite frameworks can give rise to emergent optical properties, including photoluminescence features beyond those of the host lattice.^41–44^ The solution-processed nature of these films renders them susceptible to non-uniform crystallization, which can give rise to spectral heterogeneity arising from various emissive domains within a single film.^45,46^ Herein, we present a systematic investigation of manganese­(II) (Mn)-doped chiral R-(+)- or S-(−)-1-(1-naphthyl)­ethylammonium lead bromide, (R/S-NEA)_2_PbBr_4_ perovskite thin films and demonstrate that the presence of multiple emissive components gives rise to a bisignate CPL line shape that is intrinsic to the ensemble-averaged chiroptical response of the film. Collectively, these studies highlight the broader importance of accounting for domain-specific CPL contributions in solution-processed thin films and may offer design strategies for engineering thin films with well-defined CPL spectra.

## 2 Methods and Materials

### Materials

Lead bromide (PbBr_2_) (99.99% trace metals basis), N-dimethylformamide (DMF) (98%), dimethyl sulfoxide (DMSO) (99.8%), and R-(+)- or S-(−)-1-(1-naphthyl)­ethylamine (R/S-NEA) (≥99%) were purchased from Millipore Sigma. Manganese chloride (MnCl_2_) (99%) and hydrobromic acid (48% w/w aqueous solution) were purchased from Thermo Fisher Scientific. Ethanol (200 proof), chlorobenzene (ACS 99.5%), and toluene (ACS grade) were purchased from Fisher Scientific.

### Synthesis of R/S-NEABr

R/S-NEABr was synthesized by adding 1 mL (6.2 mmol) of R/S-NEA to 15 mL of ethanol in a round bottom flask placed in an ice bath. Once the temperature reached 0 °C, 0.7 mL (6.2 mmol) of hydrobromic acid was added dropwise via syringe, and the reaction was stirred for 2 h. The solvent was removed by rotary evaporation under vacuum at 40 °C to yield a pale orange solid. The crude solid was dissolved in a minimal volume of chlorobenzene with sonication and transferred to a beaker for overnight recrystallization. The resulting orange crystals were collected by vacuum filtration and washed with chilled toluene; this process was repeated until the filtrate became colorless. The final product was then dried under vacuum prior to use.

### Preparation of Thin Films

To prepare chiral (R/S-NEA)_2_PbBr_4_ thin films, R- or S-NEABr and PbBr_2_ were dissolved in DMF/DMSO (6:4 v/v) at a 2:1 molar ratio. Indium tin oxide (ITO) substrates were sequentially ultrasonicated in acetone (5 min) and ethanol (10 min), rinsed thoroughly with deionized water, blown dry, and ozone-plasma cleaned for 12 min. All film deposition steps were carried out inside an inert atmosphere glovebox. The precursor solution (100 μL) was then deposited onto a substrate during spin-coating at 5000 rpm for 30 s. Toluene was used as an antisolvent wash and dispensed ∼10 s after the onset of spinning. The as-deposited films were subsequently annealed at 130 °C for 60 min in a vacuum oven to induce crystallization. Note that preparation of precursor films (S/R_precur_) was made the same way, but without annealing. To prepare Mn-doped films a 1:10 Mn:Pb ratio was used as the precursor solution and the same spin coating/antisolvent wash were employed. Here, however, a 120 min annealing step was performed.

### Characterization

UV-visible absorbance spectra were collected using an Agilent Cary 60 UV-Vis spectrometer and circular dichroism (CD) spectra were collected using a JASCO J-810 spectropolarimeter. Steady-state photoluminescence (PL) and photoluminescence excitation (PLE) spectra were recorded using a Horiba Jobin Yvon Fluoromax-3 fluorescence spectrometer and CPL spectra were collected using an OLIS CPL Solo UV/Vis spectrometer in reflectance mode, with a 90° geometry. The sample was mounted at 15° to maximize photoluminescence collection efficiency, and a 370 nm LED light source was used for excitation. Optical micrographs and area-specific absorbance spectra were acquired using a CRAIC 2030 PV Pro UV-visible microspectrophotometer and XRD patterns of the chiral films were characterized by a Bruker powder X-ray diffractometer.

## 3 Results and Discussion

Chiral Mn doped (R/S-NEA)_2_PbBr_4_ hybrid organic inorganic perovskite thin films, R_HOIP_:Mn and S_HOIP_:Mn, were prepared with slight modification following previously published protocols;^7^ see Methods Section for details. Figure 1a presents absorbance (solid line) and normalized steady-state photoluminescence (PL, dashed line) spectra of an R_HOIP_:Mn thin film excited with 370 nm light. Corresponding spectra for S_HOIP_:Mn films are provided in Figure S1. The absorbance feature at ∼385 nm corresponds to the first excitonic transition of the perovskite and the emission at ∼610 nm is attributed to Mn^2+^ emission, consistent with previous works.^43^ Circular dichroism (CD) and circularly polarized luminescence (CPL) spectra of R_HOIP_:Mn (black) and S_HOIP_:Mn (red) thin films are shown in Figure 1b and 1c, respectively. Note CPL spectra were also acquired with 370 nm excitation. Mirror-image Cotton effects are observed at the first excitonic transition in the CD spectra and imply chiral induction. The Mn^2+^ emission gives rise to a strong CPL signal, with maximum *g*_*lum*_ values of 0.043 and −0.026 for the R- and S-HOIPs, respectively. Interestingly, additional spectral features are also observed at sub 550 nm wavelengths that are opposite in sign relative to that of the manganese dopant. The difference in *g*_*lum*_ magnitude between the films is attributed to the crystallization kinetics inherent to solution-processed thin films, where batch-to-batch variations in domain distribution are well-documented sources of variability;^47,48^ the opposite signs and consistent spectral line shapes confirm that the intrinsic chiroptical properties of the two enantiomers are mirror-image in nature.

**Figure 1:**
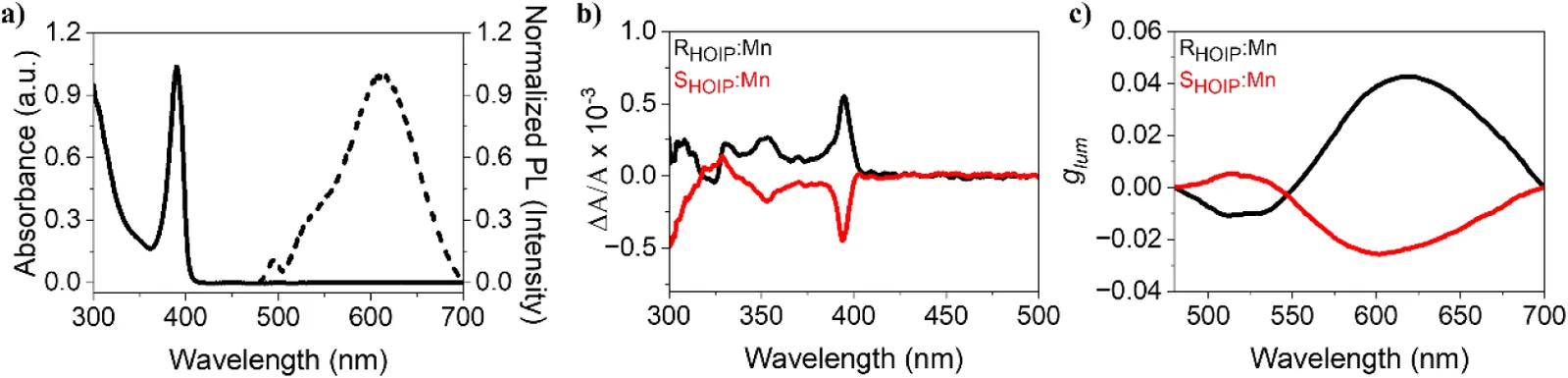
a) UV-Vis absorbance (solid line) and PL spectra (dashed line) of R_HOIP_:Mn. b) CD spectra and c) CPL spectra of R_HOIP_:Mn (black) and S_HOIP_:Mn (red) thin films.

Artifact-induced CPL signals have been shown to present as anomalous spectral features in previous works and arise from coupling of fluorescence anisotropy (FA) and linear birefringence (LB) with macroscopic structural anisotropies of the film.^28^ The mirror-image CPL spectra of the R- and S_HOIP_:Mn films (Figure 1c) confirm that instrumental artifacts arising from the 15° measurement geometry do not contribute systematically to the observed chiroptical signal. To determine if sample-anisotropy related artifacts are contributing to the observed CPL, standard measurement protocols were employed;^27^ (*i*) front and back acquisition (Figure 2a) and (*ii*) sample rotation measurements (Figure 2b).

**Figure 2:**
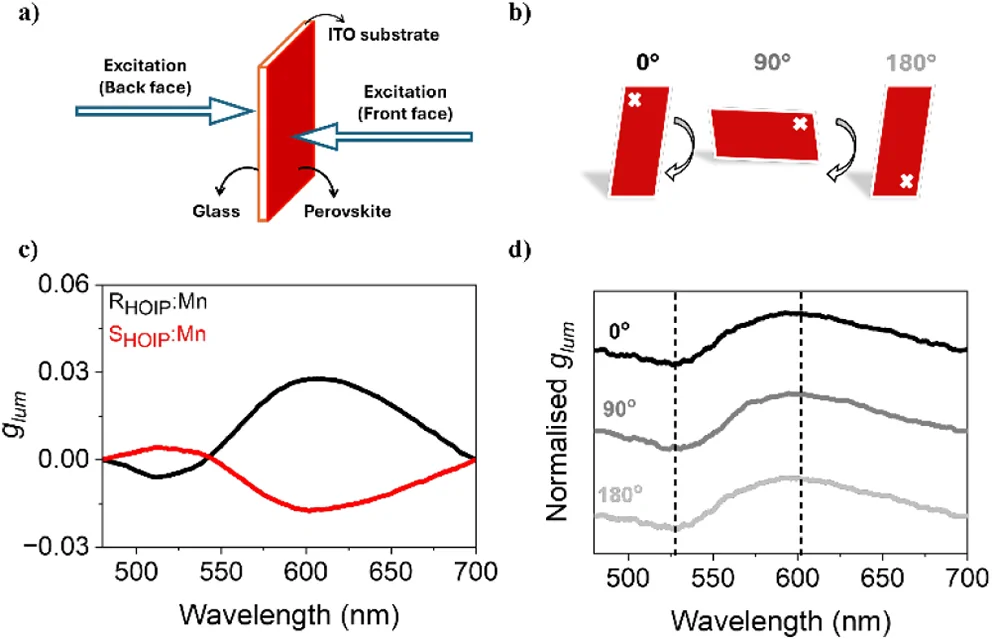
Schematic diagram of the measurement sequence used to determine artifact contributions to CPL by exciting from the front and back face of the film (a) and by rotation about the optical axis of the sample (b). Panel c) shows front and back averaged CPL spectra of R_HOIP_:Mn (black) and S_HOIP_:Mn (red) perovskite thin films and Panel d) shows rotation dependent CPL spectra of R_HOIP_:Mn thin films (black). Note that the data are offset for clarity and the dashed lines represent a guide to the eye.

LB contributions to CPL manifest as a change in signal intensity for a sample excited from the front and back face. Averaging the front and back face spectra therefore represents the true CPL signal from sample-anisotropy related contributions.^20^ Figure 2c shows the average CPL spectra of R_HOIP_:Mn (black) and S_HOIP_:Mn (red) thin films deposited on ITO collected from the front and back face. Note, the corresponding PL spectra are shown in Figure S2a. The persistence of the bisignate feature confirms that the spectral response is not rooted in linear anisotropy-based artifacts. These conclusions are corroborated by studies performed by rotating the sample about its optical axis. True CPL signals remain invariant with rotation angle, whereas FA and LB coupling often lead to spectral shifts.^19^ Figure 2d shows CPL spectra of a R_HOIP_:Mn at 0°, 90°, and 180° and the corresponding PL spectra are shown in Figure S2b. The dashed lines at ∼525 nm and ∼605 nm highlight only a minor peak shift across the three angles and indicates near negligible sample anisotropy related artifact contributions.

Next, we assess whether the observed CPL features are associated with emission from other photophysical processes (Figure 3). Panel a) presents a PL spectrum (black) of a R_HOIP_:Mn thin film fit to a sum of three Gaussian peaks centered at 495 nm (light blue), 538 nm (blue), and 612 nm (dark blue). The 3 peak positions correspond to the spectral maxima and shoulders resolved in the broad PL line shape with all 3 components required to adequately reproduce the overall PL envelope shown as an orange dashed line. A least squares fit of the data to the CPL spectrum was then performed in which the peak position and FWHM, but not its intensity or sign, were constrained; the corresponding fitting parameters are summarized in Table S1. Figure 3b plots the result of the least squares fitting and Figure 3c overlaps the envelope (pink) to the CPL spectrum (black). The shape of the CPL spectrum is qualitatively reproduced and suggests that the negative *g*_*lum*_ observed between 480–550 nm originates from spectral contributions at 495 and 538 nm. Similar results are observed for S_HOIP_:Mn thin films and are shown in Figure S3 along with the fitting parameters in Table S2. These findings are corroborated by photoluminescence excitation spectra of the R_HOIP_:Mn film collected at 495, 540, and 610 nm; see Figure S4. For all three wavelengths, the emission features are populated through excitation of the perovskite.

**Figure 3:**
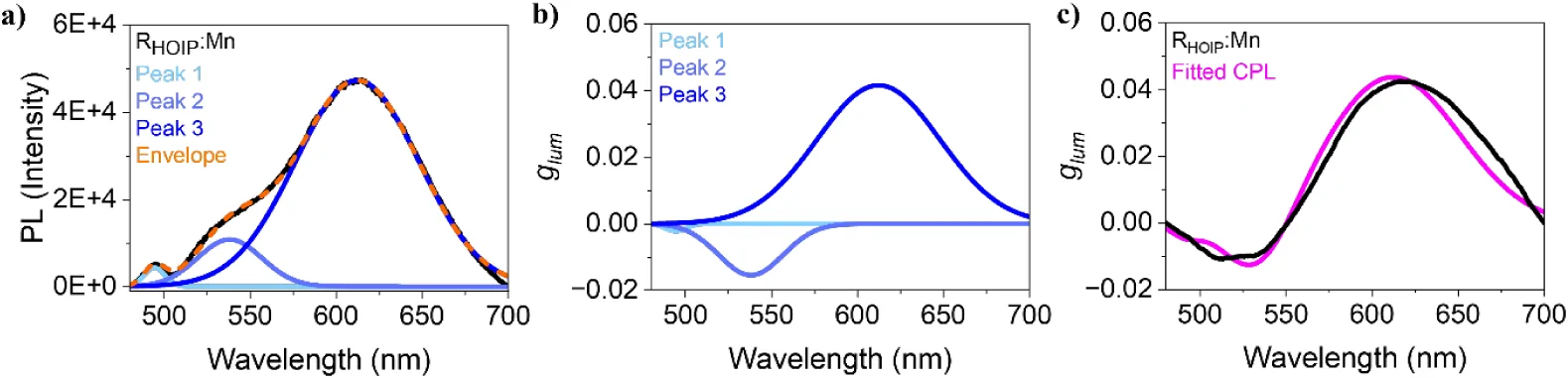
(a) PL spectrum of R_HOIP_:Mn thin film (black) fit to a sum of three peaks (light to dark blue). The envelope to the fitting is shown as an orange dashed line. (b) Intensity and sign of the three peaks following a least squares fitting to the CPL spectrum. (c) Comparison of the summation (envelope) of the three peaks in Figure 2b (pink) to the CPL spectrum (black).

To determine the origin of the spectroscopic features, we then performed control experiments on undoped perovskites (R_HOIP_) and reaction precursors prior to annealing (R_precur_); see Methods Section for additional details on the preparation of the thin films. X-ray diffraction (XRD) patterns of the three films (Figure S5) indicate similar crystallinity for R_HOIP_ and R_HOIP_:Mn, whereas no diffraction peaks were observed for R_precur_. Figure 4 plots the absorbance (a), CD (b), and PL spectra (c) of R_precur_ (purple), R_HOIP_ (green, dashed line), and R_HOIP_:Mn (black) thin films. The absorbance and CD spectra of R_HOIP_ and R_HOIP_:Mn thin films is nearly identical, whereas R_precur_ thin films exhibit a distinct absorbance feature at ∼320 nm with corresponding Cotton effects between 310 and 330 nm. Notably, the PL spectra of the R_precur_ and the R_HOIP_ show two emission features, at ∼495 and ∼538 nm, consistent with that observed for R_HOIP_:Mn. Interestingly, weak but negative CPL signals are observed for both R_precur_ and R_HOIP_ in this spectral region (Figure S6) and suggest that the opposite-sign CPL feature for R_HOIP_:Mn originates from emissive states emanating from the host perovskite framework or residual unreacted precursor. We hypothesize that the R_HOIP_:Mn and S_HOIP_:Mn thin films comprise domains with discrete transitions responsible for the complex CPL behavior, supported by the independent spectroscopic reproduction of these features in compositionally distinct bulk films.

**Figure 4:**
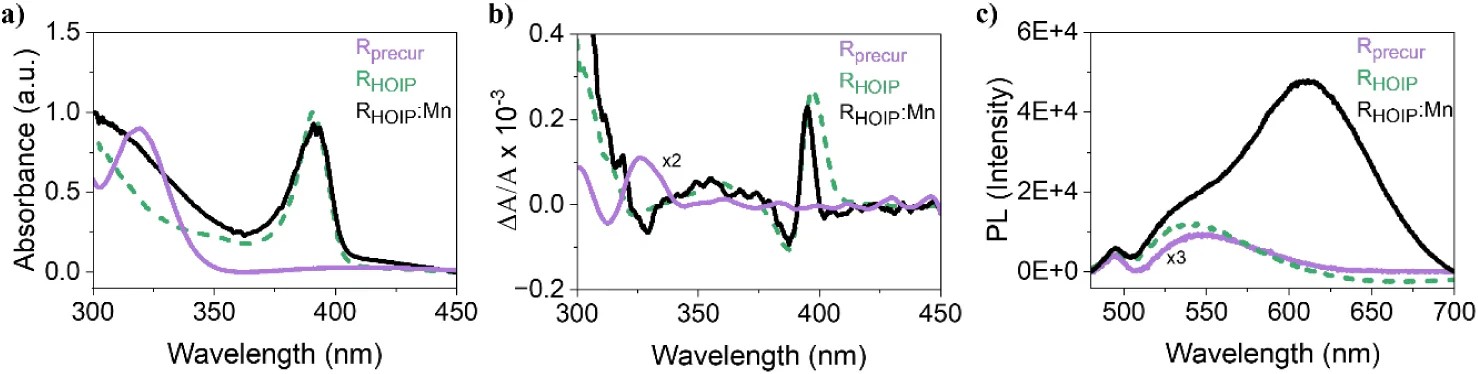
a) UV-Vis absorbance, b) CD, and c) PL spectra of R_precur_ (purple), R_HOIP_ (green, dashed), and R_HOIP_:Mn (black) thin films. For clarity, the CD and PL spectra of the precursor complex film are scaled by factors of 2 and 3, respectively.

To validate this supposition, UV-visible microspectrophotometry was employed to spatially resolve area-specific absorbance features. Figure 5 presents representative data for a R_HOIP_:Mn thin film, in which three spatially distinct domains are identified; a crystalline-like perovskite region with periodic structure (perovskite, top), a less crystalline region of perovskite material (intermediate, middle), and a region dominated by unreacted precursor (precursor, bottom). The outset of the three distinct regions shows the corresponding absorbance spectrum and reflects the absorbance of R_HOIP_, R_HOIP_ + R_precur_, and R_precur_, respectively; see Figure S7 for overlaid spectra with bulk, thin film absorbance measurements. Collectively, the spatially distinct absorbance profiles of each domain directly correspond to the emissive components identified in Figure 3, establishing their coexistence as compositionally distinct regions within the R_HOIP_:Mn and S_HOIP_:Mn thin films which are responsible for the bisignate CPL spectral features observed in Figure 1c.

**Figure 5:**
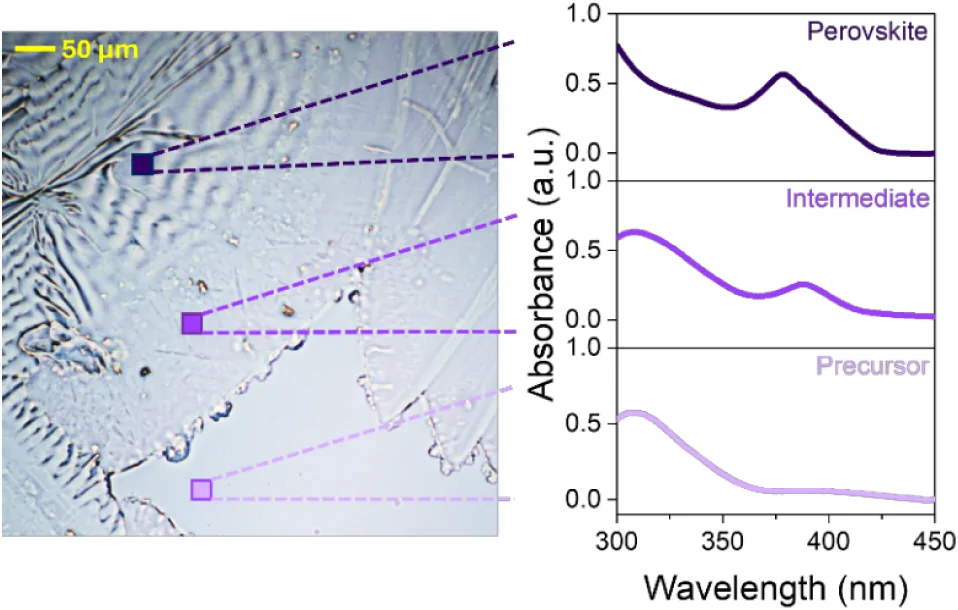
Optical micrograph (left) of a R_HOIP_:Mn thin film. The outset shows absorbance measurements of three distinct regions of the film; a periodic crystalline perovskite region (perovskite, top), a less crystalline perovskite region with excess precursor (intermediate, middle), and a region dominated by unreacted precursor (precursor, bottom).

## 4 Conclusion

This work highlights the importance of accounting for coexisting emissive contributions when evaluating CPL spectra of solution-processed thin films. Through a series of measurements on Mn-doped chiral perovskite thin films, we demonstrate that the bisignate CPL line shape arises from the coexistence of multiple spectrally distinct emissive components within the film, each contributing independently to the observed chiroptical signal. Standard artifact controls experiments, e.g., front and back face excitation and angle-dependent sample rotation, confirming that this bisignate feature is intrinsic to the film and does not arise from sample-anisotropy related effects. Control experiments on undoped chiral perovskite and precursor films and UV-visible microspectrophotometry measurements establish that the additional spectral contributions are associated with the coexistence of crystalline perovskite, intermediate, and precursor phase domains, each harboring discrete emissive states that collectively shape the observed CPL spectrum. Note that, micro-PL and micro-CPL measurements, that spatially map the chiroptical contributions across the individual domains alongside cross-polarized optical microscopy as a complementary tool for providing enhanced optical contrast, would more concretely support these conclusions, and will be the focus of future studies. More broadly, these findings serve as a cautionary note for the interpretation of CPL spectra of solution-processed thin films across material systems, emphasizing the need for spatially resolved measurements and rigorous spectral deconvolution alongside standard artifact controls to appreciate the true CPL response of a material.

## Supplementary Material



## Notes

Tables of fitting parameters with errors, UV-visible absorbance and PL spectra of S_HOIP_:Mn, PL spectra associated with anisotropy-based artifact measurements of films, least-squares fitting analysis of S_HOIP_:Mn, XRD patterns of R_HOIP_:Mn, R_HOIP_, and R_precur_, PLE spectra of R_HOIP_:Mn, CPL spectra of R_HOIP_ and R_precur_ films, comparison of absorbance spectra between localized domains and corresponding bulk films (PDF)

## #

D.H.W. deceased.
