# Supplementary material for: Spectral Contributions to Circularly Polarized Luminescence beyond Artifacts in Solution-Processed Thin Films
Source: J Phys Chem C Nanomater Interfaces. 2026 Sep 11;130(38):13232–7. doi: 10.1021/acs.jpcc.6c05545 (PMC13618029; doi:10.1021/acs.jpcc.6c05545)
Supplement: Supplementary file 1 [file jp6c05545_si_001.pdf]

Supplementary information for:

Spectral Contributions to Circularly Polarized Luminescence Beyond Artifacts in Solution-Processed Thin Films

Sithara R. Rao, [1] Justin J. O'Neil,[1] David H. Waldeck, [1#] Brian P. Bloom[1*]

[1]Department of Chemistry, University of Pittsburgh, PA 15260, USA

[*]corresponding author email: bpb8@pitt.edu

[#]deceased

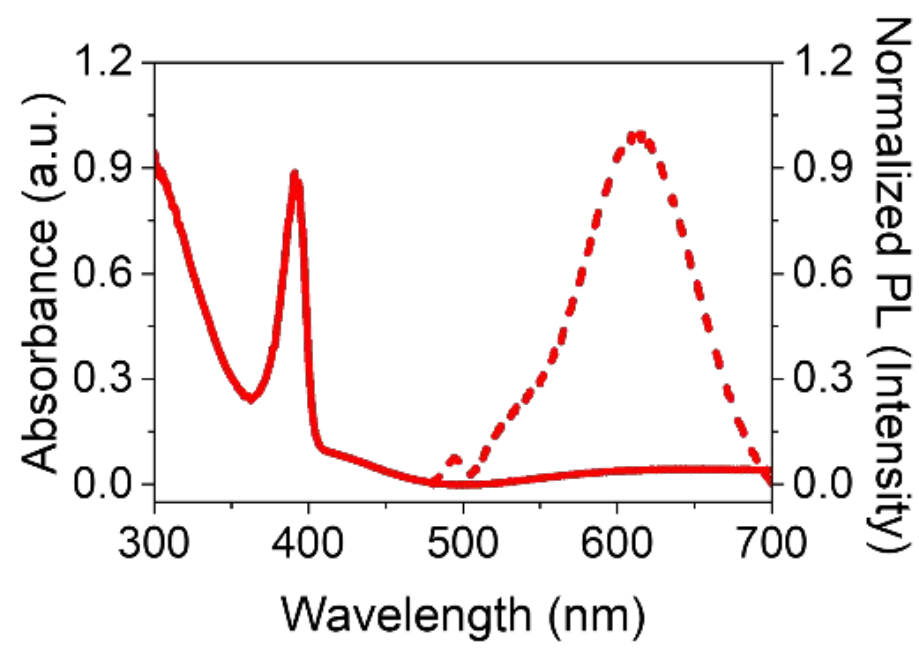

Figure S1. UV-Vis absorbance (solid line) and steady-state photoluminescence spectra (dashed line) of a S_{HOIP}:Mn thin film.

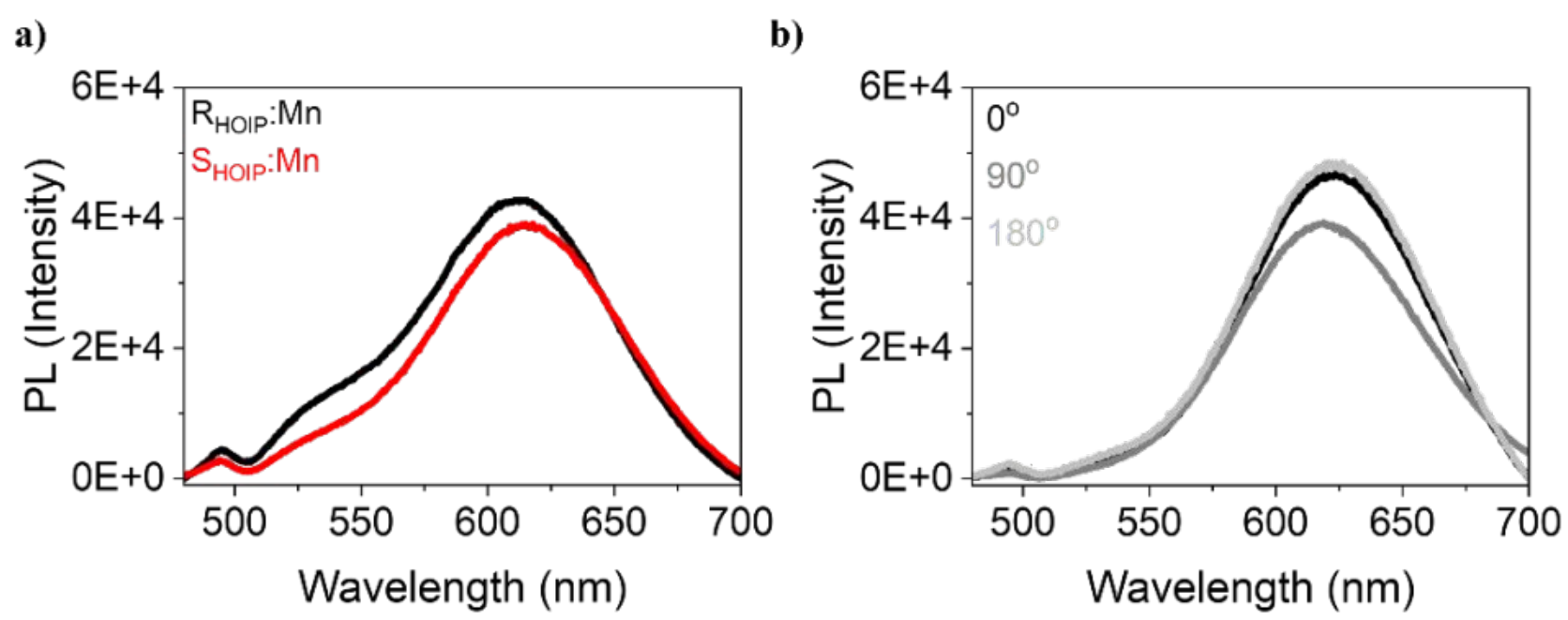

Figure S2. a) Front- and back-face averaged steady-state photoluminescence (PL) spectra of R_{HOIP}:Mn (black) and S_{HOIP}:Mn (red) perovskite thin films. b) Angle-dependent PL spectra of a R_{HOIP}:Mn thin film collected at substrate rotation angles of 0˚, 90˚, and 180˚.

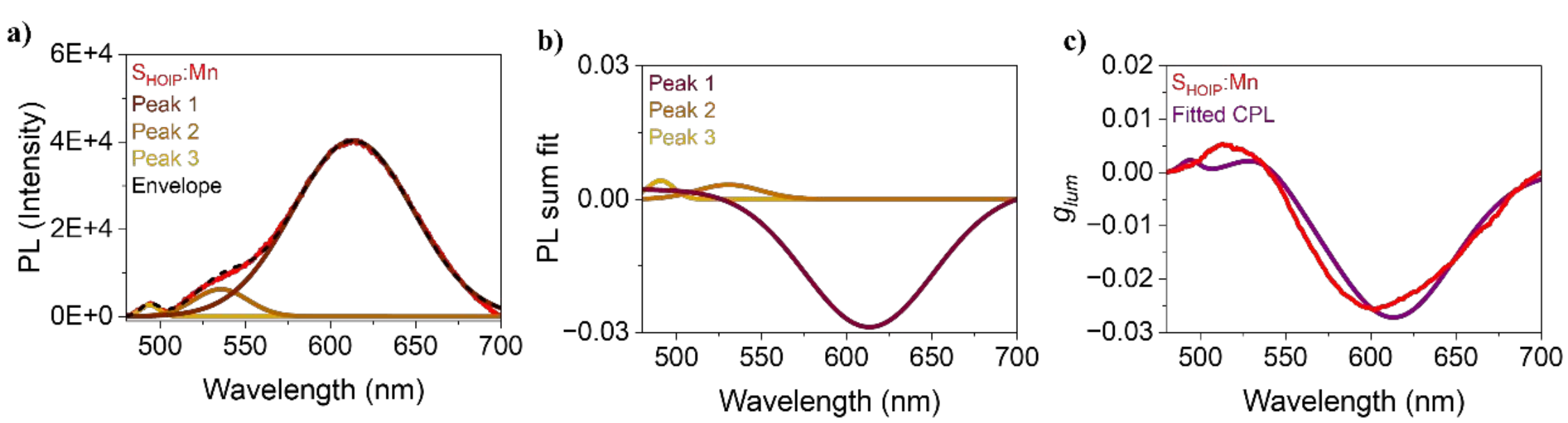

Figure S3. (a) PL spectrum of an S_{HOIP}:Mn thin film (red) fit to a sum of three peaks (light to dark brown). The envelope to the fitting is shown as a black dashed line. (b) Intensity and sign of the three peaks following a least square fitting to the CPL spectrum. (c) Comparison of the summation (envelope) of the three peaks in Figure 2b (purple) to the CPL spectrum (red).

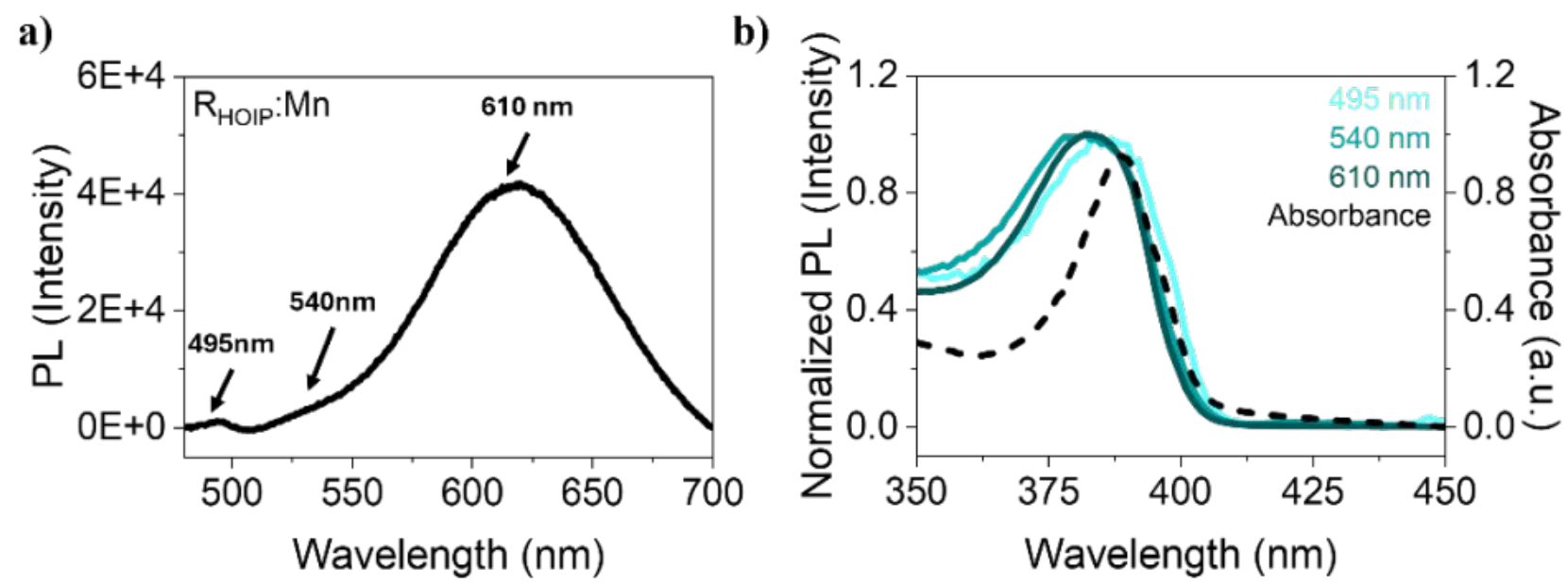

Figure S4. a) PL spectra of a R_{HOIP}:Mn thin film with arrows indicating emission wavelengths at 495, 540, and 610 nm, and b) corresponding photoluminescence excitation spectra collected at the same wavelengths. The PLE spectra overlaps well with the absorbance spectrum of the R_{HOIP}:Mn film (black) at the first excitonic transition.

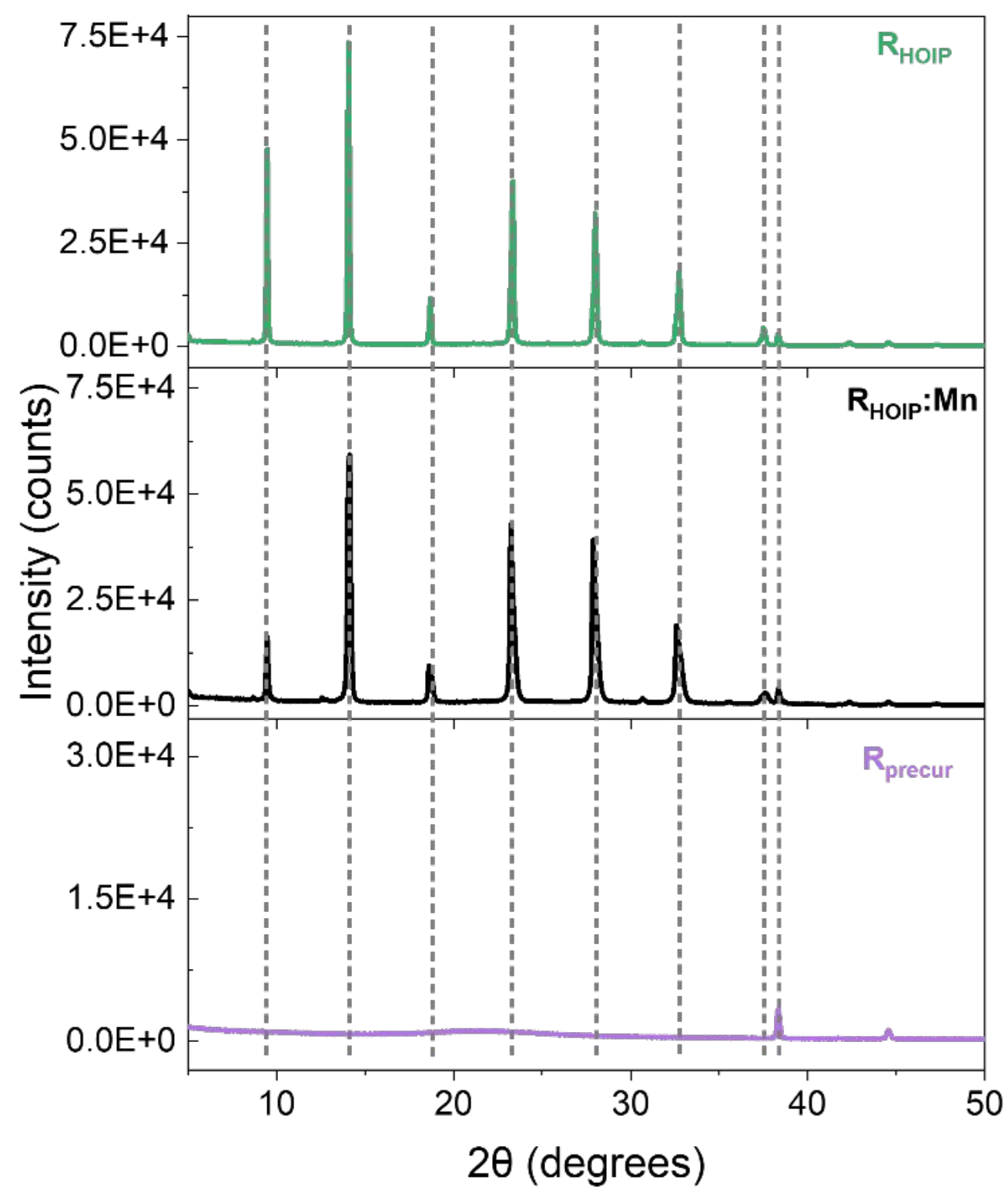

Figure S5. XRD patterns for (a) undoped R_{HOIP} (top, green), (b) R_{HOIP}:Mn (middle, black), (c) R_{precur} (bottom, purple). Vertical dashed lines indicate peaks due to <00l> planes of the perovskite, where l=4,6,8, etc. and increases with increasing value of 2θ.[1]

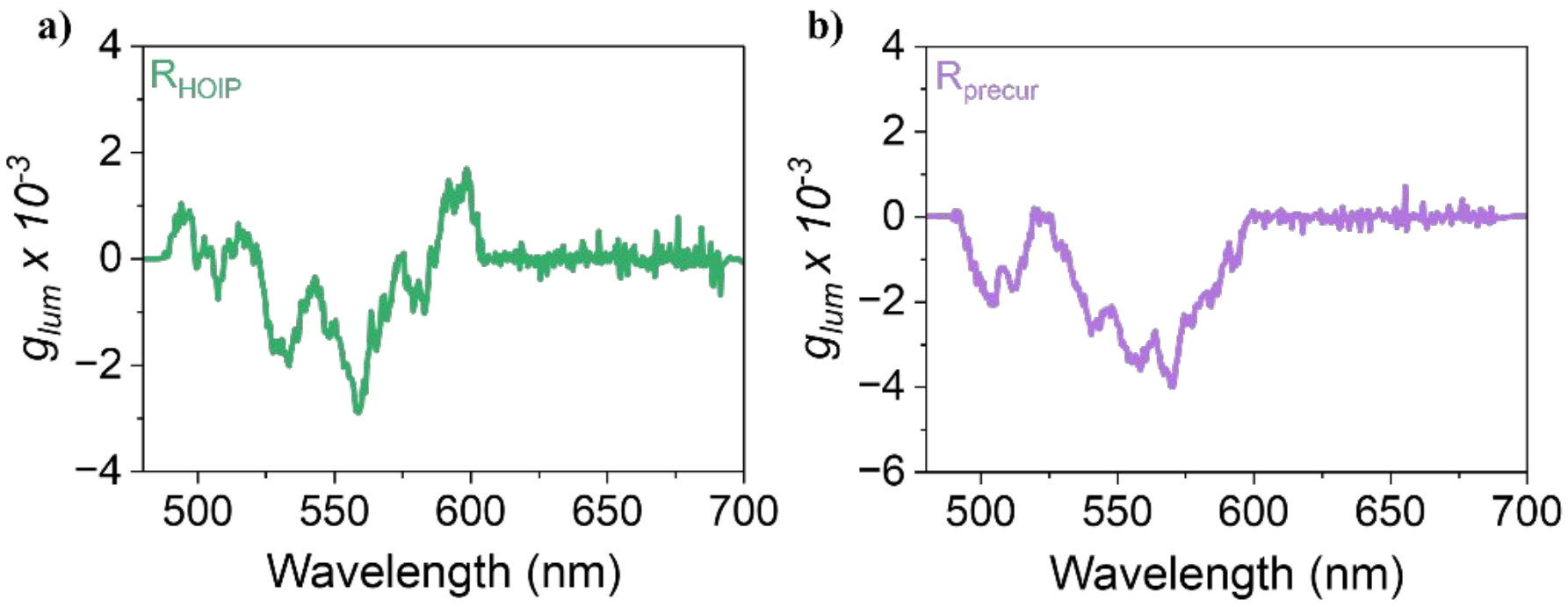

Figure S6. CPL spectra of R_{HOIP} (a) and R_{precur} (b) films exhibiting weak negative CPL signals.

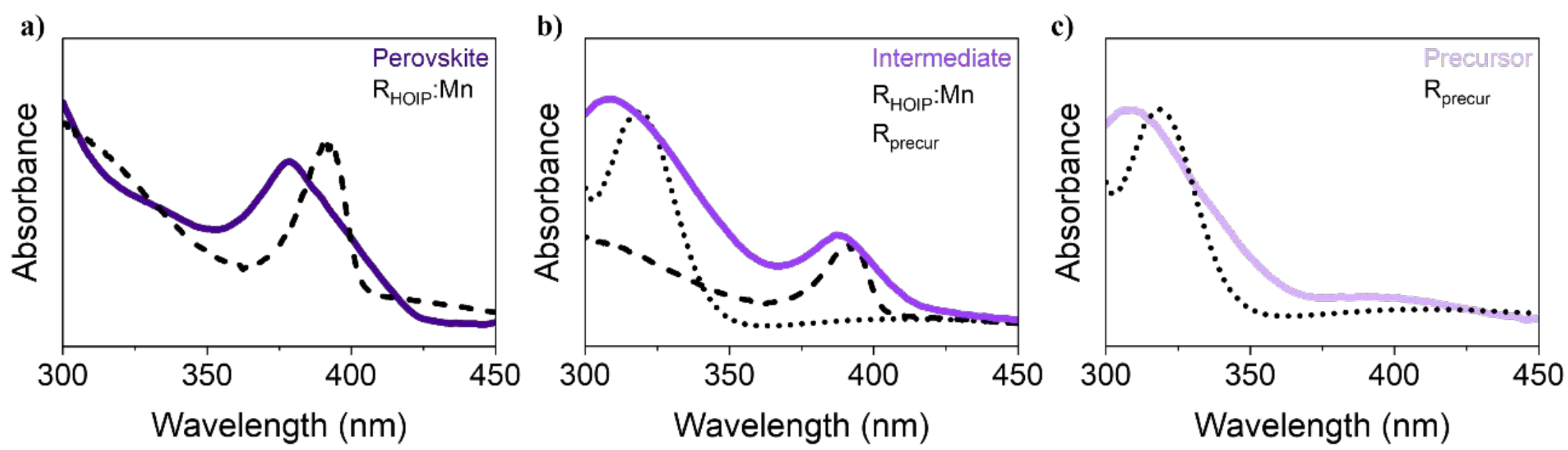

Figure S7. Absorbance spectra of three distinct domains within a R_{HOIP}:Mn thin film measured using UV–Vis microspectrophotometry, overlaid with corresponding film-scale absorbance spectra obtained from UV–Vis measurements: (a) perovskite domain (dark purple) compared with the R_{HOIP}:Mn film (black, dashed), (b) intermediate domain (purple) compared with R_{HOIP}:Mn (black, dashed)+R_{precur} (black, dotted) films, and (c) precursor domain (light purple) compared with the unannealed R_{precur} film (black, dotted). Note that slight spectral shifts and broadening in the absorbance measured through the microspectrophotometer is associated with the optical components of the instrument. The close spectral agreement demonstrates that the localized domain absorbance closely reflects the corresponding bulk film optical characteristics.

Model	**Gauss**		
Equation	y=y0 + (A/(w*sqrt(pi/2)))*exp(-2*((x-xc)/w)^2)		
Plot	Peak1($(RNEA)_2PbBr_4$ Mn doped)	Peak2($(RNEA)_2PbBr_4$ Mn doped)	Peak3($(RNEA)_2PbBr_4$ Mn doped)
y0	**0 ± 0**	**0 ± 0**	**0 ± 0**
xc	**494.5 ± 0**	**538.16366 ± 0.27028**	**612.35602 ± 0.10114**
w	**12 ± 0**	**37.52039 ± 0.51563**	**71.64624 ± 0.20888**
A	**65000 ± 0**	**506751.18068 ± 9297.64395**	**4247992.79095 ± 10802.6633**
R-Square (COD)	**0.99837**		
Adj. R-Square	**0.99835**		

Table S1. Gaussian fitting parameters obtained from the least-squares deconvolution of the PL spectrum of the R_{HOIP}:Mn thin film, including peak center (xc), peak width (w), and peak area (A). An R^2 value of 0.998 confirms the quality of the fit.

Model	Gauss		
Equation	y=y0 + (A/(w*sqrt(pi/2)))*exp(-2*((x-xc)/w)^2)		
Plot	Peak1(($(SNEA)_2PbBr_4$ Mn doped)	Peak2(($(SNEA)_2PbBr_4$ Mn doped)	Peak3(($(SNEA)_2PbBr_4$ Mn doped)
y0	**0 ± 0**	**0 ± 0**	**0 ± 0**
xc	**494 ± 0**	**538.70483 ± 0.28697**	**612.33789 ± 0.0632**
w	**11 ± 0**	**36 ± 0**	**70.55774 ± 0.12745**
A	**36500 ± 0**	**250000 ± 0**	**3557246.35276 ± 5534.89556**
R-Square (COD)	**0.99834**		
Adj. R-Square	**0.99833**		

Table S2. Gaussian fitting parameters obtained from the least-squares deconvolution of the PL spectrum of the S_{HOIP}:Mn thin film, including peak center (xc), peak width (w), and peak area (A). An R^2 value of 0.998 confirms the quality of the fit.

[1] Du, K. Z.; Tu, Q.; Zhang, X.; Han, Q.; Liu, J.; Zauscher, S.; Mitzi, D. B., Two-Dimensional Lead(II) Halide-Based Hybrid Perovskites Templated by Acene Alkylamines: Crystal Structures, Optical Properties, and Piezoelectricity. *Inorg. Chem.* **2017,** *56* (15), 9291-9302.
